# Possible Association of *APOE* Genotype with Working Memory in Young Adults

**DOI:** 10.1371/journal.pone.0135894

**Published:** 2015-08-19

**Authors:** Lindsey I. Sinclair, Katherine S. Button, Marcus R. Munafò, Ian N. M. Day, Glyn Lewis

**Affiliations:** 1 School of Social & Community Medicine, University of Bristol, Bristol, United Kingdom; 2 MRC Integrative Epidemiology Unit (IEU) at the University of Bristol, Bristol, United Kingdom; 3 Division of Psychiatry, University College London, London, United Kingdom; 4 UK Centre for Tobacco and Alcohol Studies, School of Experimental Psychology, University of Bristol, Bristol, United Kingdom; Duke University, UNITED STATES

## Abstract

**Background:**

Possession of the ε4 allele of the Apolipoprotein E *(APOE)* gene is associated with an increased risk of Alzheimer’s disease. Early adult life effects of ε4 are less well understood. Working memory has been relatively little studied (compared to episodic memory) in relation to *APOE* genotype despite its importance in cognitive functioning. Our hypothesis was that ε4 would lead to an impairment in working memory in young adults.

**Methods:**

We studied working memory using a computerised n-back task in the Avon Longitudinal Study of Parents and Children (ALSPAC) at age 18. Data was available for 1049–1927 participants and for the 2- and 3-back versions of the task. Using multiple and multi-level regression controlling for important confounders we examined the association between *APOE* genotype on accuracy and reaction times.

**Results:**

There was no evidence of a genotype effect on accuracy when the two difficulty levels were examined separately. There was some evidence to support a deleterious effect of the ε4 allele on n-back accuracy in the multi-level regression. There was weak evidence that the ε22 group were less accurate but the numbers were very low in this group. The ε34 group had faster reaction times than the reference ε33 group in all adjusted analyses but the ε44 group were only faster in the 3-back condition in multi-level analyses.

**Conclusions:**

There was no evidence of benefit in ε4 carriers, but there was some evidence of a detrimental effect on working memory in this large study.

## Introduction

*APOE* is a gene found on chromosome 19 in humans, which encodes a protein (ApoE) involved in lipid transport. ApoE is the main lipid transport protein in the brain. There are 3 known variants of *APOE*: ε2, ε3 and ε4. E4 is thought to be the ancestral allele of *APOE*, [1] but in the UK population the reported frequencies of ε3, ε4 and ε2 are 0.78, 0.14 and 0.08 respectively.[2] These variants result from single nucleotide polymorphisms at two locations: T2060C(Cys112Arg) and C2198T (Arg158Cys).[3] The ε3 allele has cysteine and arginine in these two positions, the ε2 allele has cysteine in both and the ε4 allele has arginine in both.[4] Individuals carry two copies of *APOE* and thus may be heterozygous or homozygous (e.g. ε33, ε34).

Possession of an ε4 allele leads to an increased risk of late onset Alzheimer's disease (LOAD). [5] One ε4 allele confers a threefold increase in risk and possession of two ε4 alleles confers a tenfold increase in risk.[6] It is now known that ApoE is involved in neuronal repair, with E4 being the least efficient isoform [7] and that *APOE* genotype influences outcome following head injury.[8]

Although episodic memory is the major cognitive process affected early in LOAD, it is not the only process affected early in the the disease process.[9] Poor working memory performance has been reported as one of the earliest deficits seen in Alzheimer's disease.[10] Deficits in working memory have also been demonstrated in mild cognitive impairment, considered to be a prodrome for LOAD.[11] There are several different types of working memory, for example spatial and verbal. These are also thought to have separate storage and maintenance processes.[12] Different working memory tasks measure different aspects of working memory, which can make it difficult to compare studies.

The relationship between working memory and ε4 has been relatively little studied. There are suggestions from the quantitative results of a RVIP (rapid visual information processing) task reported in a small fMRI study of 41 young adults that ε4 may be beneficial for attention but there was no adjustment for confounding factors. [13] Conversely studies of middle aged and older adults have found a deleterious effect of ε4 on working memory. [14–16] A study of 445 people of mixed ages found no evidence for an effect. [17]

The possibility of the ε4 allele having positive effects earlier in life (positive pleiotropy) has been investigated over the last 10 years with mixed results (e.g. [18, 19]). There is no association between ε4 and IQ at age 8 years [20], but ε4 allele posession has been shown to have a deleterious effect on IQ at age 80 including in non-demented participants.[21] Several fMRI studies have reported reduced hippocampal activation in young adult e4 carriers. [22–24] For example, Mondadori and colleagues found in an fMRI study of 34 subjects that those with the ε34 genotype decreased their hippocampal activation with increasing number of trials of an episodic memory task, whereas those with ε32 and ε33 increased their activation. They suggested that this reflected more efficient learning in ε34 carriers, arguing that those with the ε34 genotype required less hippocampal activation for the same memory performance.

In this study we aimed to study the relationship between *APOE* genotype and working memory assessed using the n-back working memory task in a large community sample of young adults aged 18 years. We hypothesised that *APOE* genotype would be associated with working memory performance, with the worst performance being in ε4 carriers in a dose dependent manner due to its lower efficiency in neuronal repair.

The Avon Longitudinal Study of Parents and Children (ALSPAC) (http://www.bristol.ac.uk/alspac) is a prospective study which was set up in 1991. [25–27] This large sample permitted the inclusion of many more ε4 and ε2 homozygotes than in previous studies. No previous study has included more than 500 participants and all have had small numbers of ε44 homozygotes. For example, in the study by Reinvang and colleagues there were only 13 participants with an ε44 genotype.[14]

## Methods & Materials

### Sample

The Avon Longitudinal Study of Parents and Children (ALSPAC) (http://www.bristol.ac.uk/alspac) is a prospective study which was set up in 1991. [25–27] All pregnant women in the former Avon area with an expected date of delivery between April 1991 and December 1992 were eligible for inclusion. Recruitment was via a wide range of methods. In total 14,541 women were enrolled, resulting in 14,062 live births and 13,988 children alive at one year. For reasons of confidentiality data on the 13 triplet and quadruplet children were not available for analysis. At age 7, a further 548 eligible children and after age 8 a further 452 children were added to the sample when their mother enrolled, giving a total sample size of 15,247 eligible pregnancies and 14,775 live births. Data were collected from self-report questionnaires, teacher report questionnaires, medical, educational and other records, birth registries, and hands on assessment. Detailed information has been collected since birth via questionnaires and at annual clinics. Ethical approval for the main study was obtained from the ALSPAC ethics and law committee and the local research ethics committees, as described in detail previously.[28] Written informed consent was provided by the parents and then from the young people when they reached the age at which they were able to provide this. As this study purely related to analysis of previously collected data, no specific ethical approval was required for this study.

### APOE Genotyping

DNA samples were available in 2009 for 7091 children, 63% of the 11343 ALSPAC children with potential DNA samples available. Genotyping of the young people for *APOE* was undertaken by integrated single label liquid phase assay as described previously.[20] Full details of this method have been published previously.[2]. PCR samples were analysed using a 384 well LightTyper instrument (Roche diagnostics GmbH) and genotypes determined using LightTyper software, Ver 1. Samples were classified as ε2/2, ε 2/3, ε 3/3, ε 3/4, ε 4/4, ε 4/2 or unknown. In total 95% of the available samples were genotyped. After exclusion of siblings and children of known non-white ethnicity, 5,995 children had genotype data. There was no strong evidence of a sex difference in genotype distribution or of a deviation from Hardy Weinberg Equilibrium. *APOE* genotypes were available for 2,099 participants with 2-back data available and 2085 with 3-back data available.

### Measures

Working memory was assessed using a computerised version of the n-back task. During this task a series of numbers (0–9) were presented on the screen. Participants were ask to respond on each occasion whether the number was the same as that presented N numbers ago, or if it was not. The numbers were presented in black on a white screen for 500ms, with 3000ms allowed for participants to respond by pressing “1” for a target and “2” for a non-target stimulus. Accuracy to targets and non-targets in the 2 and 3 back conditions was used as the primary outcome. This method of analysis accounts for response bias i.e. differential responding by participants to targets versus non-targets. For example, it would be easy for a participant to be 100% accurate to targets, if they selected targets on every trial and thus had an accuracy to non-targets of 0%. By analysing both at the same time any such bias is taken into account.

Accuracy to target was defined as the proportion of targets that had been correctly indicated. This is the equivalent of “hits” in previous literature. Accuracy therefore varied between 1 = perfect accuracy and 0 = no targets correctly indicated. Accuracy to non target was defined as the proportion of “non-targets” that the participant correctly identified. In order to make the data more readily interpretable we present accuracy as a percentage. The reaction times were calculated as the median of all the individual reaction times in each of the levels (2 or 3 back) for target or non-target trials. Thus there were 2 different original variables available at each level of the n-back: reaction time to target and reaction time to non target.

The data were collected from participants who attended the teen focus 4 clinic at approximately 18 years of age (mean 17.8 yrs, SD 0.456). Of the 5,217 participants who attended the clinic 3987 performed the N-back, 2135 of whom had an *APOE* genotype available. Three hundred and ninety one participants had missing data excluded on the 2-back and 341 on the 3-back as they gave no response to any item on the task. This resulted in a total of 1,927 individuals with useable 2-back data and an *APOE* genotype available and 1,907 individuals with useable 3-back data and an *APOE* genotype available, as shown in S1 Fig.

### Co-variates

Co-variates adjusted for included demographic variables such as sex, family home-ownership status (coded as mortgaged/owned, private rental, subsidised rental or other) and maternal education, which we expected to be associated with the outcome variable. These same variables were used in the previous study by Wardle et al who found effects on performance in the n-back. [29] In addition, as there has been some debate about whether *APOE* genotype influences IQ which is strongly associated with n-back performance we adjusted for full-scale IQ.[29] This was measured at age 8 using the 3^rd^ edition of the WISC as described in detail by Wardle et al. [29]. Other co-variates were chosen based on previous research, theory, or that they were associated with *APOE* and outcome and therefore might be a confounder.

Low density lipoprotein (LDL) and whether the participant had ever had a serious head injury were also included as co-variates. LDL is known to be affected in an allele dose dependent manner by *APOE* genotype and ApoE plays a major role in neuronal repair by re-distributing lipids to regenerating axons.[1, 30, 31] However, there are other influences on LDL level so it might act as a confounder as well as being on the causal pathway between APOE genotype and cognitive function. For this reason we decided to perform adjustments with and without LDL as a covariate. Cholesterol is important in cell membrane and myelin formation and, compared to other organs, the brain has a high cholesterol content.[32] It is currently thought that the major causal pathway between ε4 possession and cognitive dysfunction is due to protein instability, neurotoxic fragment production, increased amyloid beta production (and decreased clearance) and increased tau phosphorylation rather than being via LDL and cerebrovascular disease.[33–36] It has been shown that outcome following head injury is worse for those with an ε 4 allele and head injuries are known to affect a range of cognitive functions.[37, 38]

The variation of IQ and other co-variates with *APOE* genotype is shown in Table 1. There was no evidence to support an association of any of the co-variates with *APOE* genotype. However, we included these variables as they would be expected to improve the overall fit of the regression model and in aggregate could be confounding the relationship. We considered a number of other co-variates including alcohol use and smoking but did not include them as they did not have an association with *APOE* genotype and we had no theoretical basis for an association with the outcome.

**Table 1:** Number (%) and means (standard deviations) for demographics^1^ and other co-variates by Genotype.

Variable	22	32	33	34	44	F test or X^2^
*Gender* Male Female	6 (35.29%)11 (64.71%)	157 (49.37%)161 (50.63%)	543 (44.69%)672 (55.48%)	263 (48.52%)279 (51.48%)	16 (37.21%)27 (62.79%)	X^2^ = 5.79, p = 0.22
Full scale IQ age 8 (SD)	112.80 (10.66)	107.50 (15.68)	108.12 (15.94)	108.54 (16.49)	110.81 (15.38)	
*Mother’s highest educational qualification*CSEVocationalO levelA levelDegree	0 (0%)0 (0%)7 (46.67%)6 (40.00%)2 (13.33%)	38 (13.06%)21 (7.22%)100 (34.36%)76 (26.12%))56 (19.24%)	104 (9.29%)77 (6.88%)382 (34.14%)322 (28.78%)234 (20.91%)	36 (7.19%)36 (7.19%)163 (32.53%)150 (29.94%)116 (23.15%)	2 (5.41%)5 (13.51%)16 (43.24%)9 (24.32%)5 (13.51%)	X^2^ = 18.99, p = 0.27
*Home Ownership*Mortgage/ownedPrivate rentalSubsidised rentalOther	15 (100%)0 (0%)0 (0%)0 (0%)	247 (85.47%)13 (4.50%)18 (6.23%)11 (3.81%)	997 (88.78%)41 (3.65%)60 (5.34%)25 (2.23%)	429 (85.12%)17 (3.37%)43 (8.53%)15 (2.98%)	31 (77.50%)1 (2.50%)5 (12.50%)3 (7.50%)	X^2^ = 17.70, p = 0.13
*LDL age 9* mmol/litre (SD)	1.46 (0.76)	1.92 (0.47)	2.35 (0.55)	2.50 (0.52)	2.86 (0.63)	F = 65.19, p <0.001
*Ever unconscious following a head injury*	2 (11.76%)	23 (7.23%)	121 (9.96%)	50 (9.23%)	3 (6.98%)	X^2^ = 2.63, p = 0.62
*Alcohol use frequency*NeverMonthly or less2-4x a month>2x month	08 (50.00%)7 (43.75%)1 (6.25%)	1 (0.37%)65 (23.90%)139 (51.10%)67 (24.63%)	23 (2.32%)266 (26.87%)446 (45.05%)255 (25.76%)	13 (2.89%)115 (25.56%)212 (47.11%)110 (24.44%)	07 (17.07%)24 (58.54%)10 (24.39%)	X^2^ = 17.77, p = 0.12

^1^Data shown is for all study participants who had an *APOE* genotype available and who completed the n-back testing session. Qualifications shown are British. O levels and CSEs were taken aged 16, A levels are taken aged 18. CSEs were a lower level of qualification than O levels, which were taken by those not expected to pass O levels.

### Analyses

Accuracy on the n-back task across the 2 and 3 back levels was the primary outcome. Statistical power was estimated at 87% to detect a 3.5% difference in accuracy rates at the 2 and 3 back levels, based on data from previous analyses.[29]

In all analyses we used multi-level regression using the stata command xtreg with individual as a random effect. The ε 33 group, the most frequent genotype, was used as the reference group throughout. Initially the 2 and 3 back levels were considered separately, as some individuals were missing data from one n-back level only. For this analysis the data was re-shaped into long format according to target/non-target. This permitted the regression to model accuracy to target and non-target simultaneously, rendering separate analysis of false alarms and discriminability unnecessary. Multi-level regression has the advantage that it accounts for clustering in the data within individuals. For each outcome variable at each level of difficulty the regression was performed with *APOE* genotype as the only exposure variable to test the crude (i.e. unadjusted) association and then a further regression was then performed with adjustment for all co-variates.

Finally we used a further multi-level regression to examine the effects of *APOE* genotype on outcomes across the 2 and 3 back levels. To test for overall gene effect likelihood ratio testing was performed on the models with and without the *APOE* variable. To test for a linear effect or quadratic effect of *APOE*, genotype was included in separate regression models as a linear term and as a quadratic term. These were again performed as both crude and adjusted analyses.

In the multi-level analysis individuals with missing or missing data at either the 2 or 3 back level were excluded, which reduced the number available for analysis (n = 1834). Two hundred and eight participants had missing data for the 2-back, 184 for the 3-back and 91 had missing data in both.

We primarily used *APOE* genotype as a categorical variable, This is because there is an established literature to support alleles 2 and 4 being differently functional relative to 3. The risk of Alzheimer’s disease, however, shows an allele dose effect for the ϵ4 allele. For this reason we decided that it was sensible to analyse the data as both a categorical variable and as a linear term in order to look for allele dose effects. We performed the analysis with a quadratic term to allow for non-linearity in the results. The relationship between APOE genotype and plasma LDL cholesterol is linear[30], but the relationship between APOE and AD risk is not, as mentioned previously. For this reason we felt that it was important to include both terms in our analyses. We chose to reduce the risk of type 1 errors by using one overall significance test to test for any differences between the categories.

## Results

Of the 2,135 participants who completed the n-back and had an *APOE* genotype available, 17 had the ε22 genotype, 318 had the ε32 genotype, 1,215 had the had the ε33 genotype, 542 had the ε34 genotype and 43 had the had the ε44 genotype. There was no evidence of deviation from Hardy-Weinberg equilibrium. The raw values for accuracy and reaction times adjusted for target are shown in S1 Table.

### Accuracy

The data had been previously examined by Wardle et al who found that hits were normally distributed at the 2 and 3 back levels and that the 3-back was more difficult with lower accuracy at the 3 back level.[29] Full scale IQ was associated with accuracy at both levels of difficulty. At the 2-back level the regression coefficient was 0.003 (95% CI = 0.00273, 0.00416, p < 0.0001). At the 3-back level the regression coefficient was 0.003 (95% CI = 0.00248, 0.00374, p < 0.0001). The participants were also slightly more intelligent than the general population (mean IQ = 108.2, 95%CI = 107.5–108.9, one sample t-test against mean of 100 p value = <0.00001).

Accuracy was initially examined at the two difficulty levels separately, as shown in table 2. There was no strong evidence to support an effect of APOE genotype in the unadjusted regression at either level. In the adjusted analysis (n = 1,099) at the 2-back level, the overall p value for genotype supported there being an effect of genotype (p = 0.04), but there was no evidence to support the effect being linear or quadratic. Negative effects were seen in both the ε 22 (coefficient = -5.89%, 95% CI -15.58, 3.81) and ε 44 group (coefficient = -8.35%, 95% CI -15.51, –1.19).

**Table 2:** Regression coefficients from regressions at each level separately of overall Accuracy to target and non-target in the n-back in relation to *APOE* genotype. The results shown are for the model adjusted for target, home ownership, mother's education, IQ, gender, head injury and LDL aged 9. The ε33 genotype was used as the reference group.

*APOE* Genotype	2-back			2-back Minus LDL			3-back			3-back minus LDL		
	Co-efficient	95% CI	P value	Co-efficient	95%CI	P value	Co-efficient	95% CI	P value	Co-efficient	95%CI	P value
ε22	-5.89	-15.58 to 3.81		-5.23	-14.76 to 4.29		-0.29	-9.14 to 8.55		-0.18	-8.85 to 8.50	
ε32	1.00	-2.18 to 4.18		0.90	-1.65 to 3.46		-1.39	-0.04 to 0.02		-1.71	-3.98 to 0.56	
ε33	0			0			0			0		
ε34	0.42	-2.00 to 2.85		0.78	-1.26 to 2.83		-1.32	-3.44 to 0.79		-0.27	-2.04 to 1.51	
ε44	-8.35	-15.51 to -1.19		-7.10	-13.15 to -1.03		-5.61	-11.35 to 0.67		-2.67	-7.97 to 2.63	
Overall p value			0.04			0.04			0.25			0.48
Linear term (overall effect)	-0.80	-2.31 to 0.72	0.31	-0.31	-1.53 to 0.91	0.62	-0.01	-0.02 to -0.01	0.32	0.23	-0.84 to 1.30	0.67
Quadratic term	-0.16	-0.40 to 0.07	0.18	-0.07	-0.26 to 0.12	0.47	-0.001	-0.003 to 0.001	0.20	0.02	-0.15 to 0.18	0.85

In the adjusted regression at the 3-back level (n = 1,104) there was no strong evidence of any effect of genotype on accuracy. Although all genotype groups performed worse than the ε 33 group, there was weak evidence that this difference was greater in the ε 44 genotype group as shown in Table 2. Again there was no evidence to support a linear or quadratic effect of genotype.

When performing the multilevel regression across both levels the crude analysis did not show any evidence to support an effect of *APOE* genotype. The adjusted model showed evidence of a small decline in performance in several of the groups as shown in Table 3, including the ε22, ε34 and ε44 groups. The overall p value for genotype supported there being an effect of genotype (p = 0.0071), but there was no evidence to support a linear or quadratic association. The small decline in performance in the ε34 group was not present in the multilevel model where LDL was not included as a co-variate, but not including LDL had no effect on the relationships seen in the regressions at the separate levels. The overall variance explained by this model was 21.3% suggesting that there are many other factors involved in n-back accuracy. There was no evidence of an interaction between gender and *APOE*.

**Table 3:** Regression coefficients from multi-level regression of *APOE* genotype against overall accuracy in the n-back. Adjustments were target, home-ownership, mother's education, IQ, gender, head injury and LDL aged 9. The ε33 genotype was used as the reference group. The coefficients reflect the change in percentage accuracy.

*APOE* Genotype	Crude			Adjusted			Adjusted minus LDL		
	Co-efficient	95% CI	P value	Co-efficient	95% CI	P value	Co-efficient	95% CI	P value
ε22	0.33	-7.14 to 7.79		- 4.30	-12.2 to 3.56		-3.98	-11.71 to 3.73	
ε32	-0.21	-2.19 to 1.77		0.44	-2.12 to 2.99		0.27	-1.78 to 2.34	
ε33	0			0			0		
ε34	0.37	-1.24 to 1.97		-0.44	-2.37 to 1.48		0.36	-1.27 to 1.98	
ε44	-4.21	-8.84 to 0.42		-7.58	-13.16 to -2.00		-5.42	-10.15 to 0.70	
Overall p value			0.75			0.01			0.03
Linear term (overall effect)	-0.10	-1.05 to 0.85	0.83	-0.01	-1.95 to 0.46	0.22	-0.25	-1.23 to 0.73	0.62
Quadratic term	-0.03	-0.18 to 0.12	0.70	-0.002	-0.34 to 0.04	0.12	-0.06	-0.21 to 0.09	0.44

### Reaction Times

The same analysis strategy was then applied to reaction times. As described by Wardle et al, reaction times were slower at the 3-back level and slowed more for men than for women.[29] As the data were not normally distributed at either n-back level log transformations were used to permit parametric testing. There was weak evidence of an interaction between gender and *APOE*, which persisted in the adjusted model.

Reaction time was also initially examined at the two difficulty levels separately, as shown in table 4. In the adjusted regression at the 2-back level (n = 1099) there was statistical evidence that APOE genotype was associated with reaction time. In particular some evidence for faster reaction times was seen in the ε34 group (coefficient = -0.06, 95%CI -0.10, -0.01) and the ε32 group (coefficient = -0.05, 95%CI -0.11, 0.003). The overall p value for genotype supported there being an effect of genotype (p = 0.001), but there was no evidence to support the effect being linear or quadratic.

**Table 4:** Regression coefficients from regressions performed at each level separately of log reaction time in the n-back in relation to *APOE* genotype. The results shown are for the model adjusted for target. home ownership, mother's education, IQ, gender, head injury and LDL aged 9. The ε33 genotype was used as the reference group. The log of reaction time was used as the residuals were not normally distributed when linear regression was performed using untransformed reaction time.

*APOE* Genotype	2-back			2-back adjMinus LDL			3-back			3-back adj minus LDL		
	Co-efficient	95% CI	P value	Co-efficient	95%CI	P value	Co-efficient	95% CI	P value	Co-efficient	95%CI	P value
ε22	0.03	-0.14 to 0.21		0.05	-0.13 to 0.22		0.001	-0.22 to 0.22		0.01	-0.21 to 0.22	
ε32	-0.05	-0.11 to 0.003		-0.06	-0.11 to -0.02		-0.05	-0.12 to 0.02		-0.06	-0.12 to -0.01	
ε33	0			0			0			0		
ε34	-0.06	-0.10 to -0.01		-0.02	-0.06 to 0.02		-0.06	-0.11 to -0.01		-0.02	-0.06 to 0.02	
ε44	0.06	-0.07 to 0.19		0.06	-0.05 to 0.17		-0.06	-0.21 to 0.10		-0.04	-0.17 to 0.09	
Overall p value			0.001			0.004			0.02			0.05
Linear term (overall effect)	-0.01	-0.03 to 0.02	0.71	0.01	-0.007 to 0.04	0.19	-0.03	-0.06 to 0.01	0.11	0.01	-0.02 to 0.03	0.59
Quadratic term	-0.001	-.005 to 0.003	0.70	0.002	-0.001 to 0.006	0.232	-0.005	-0.01 to 0.0004	0.07	0.001	-0.003 to 0.005	0.77

In the adjusted analysis (n = 1104) at the 3-back level, there was again statistical evidence that APOE genotype was associated with reaction time, with evidence of faster reaction times in all groups except ε22. The overall p value for genotype supported there being an effect of genotype (p = 0.02), but there was no evidence to support the effect being linear or quadratic.

When the multi-level regression was performed, as shown in table 5, there was statistical evidence that APOE genotype was associated with reaction time in both the 2back and 3 back condition. Only the ε22 group performed worse (slower) than the ε 33 reference group. However there was no evidence for a linear relationship. The ε34 group had faster reaction times than ε33 (coefficient = -0.05, 95% CI –0.10, -0.01). This relationship was not affected by including LDL as a co-variate. The overall variance explained by this model was 4.6% suggesting that there are many other factors involved in n-back reaction times.

**Table 5:** Regression coefficients from multi-level regression of *APOE* genotype against log reaction time in the n-back with adjustment for home ownership, mother's education, IQ, gender, head injury and LDL aged 9. The ε33 genotype was used as the reference group. The log of reaction time was used as the residuals were not normally distributed when linear regression was performed using untransformed reaction time.

*APOE* Genotype	Crude			Adjusted			Adjusted minus LDL		
	Co-efficient	95% CI	P value	Co-efficient	95% CI	P value	Co-efficient	95% CI	P value
ε22	0.03	-0.13 to 0.19		0.02	-0.16 to 0.19		0.02	-0.15 to 0.19	
ε32	-0.04	-0.08 to 0.003		-0.04	-0.01 to 0.02		-0.05	-0.10 to -0.003	
ε33	0			0			0		
ε34	-0.01	-0.04 to 0.02		-0.05	-0.10 to -0.01		-0.02	-0.06 to 0.02	
ε44	0.01	-0.09 to 0.11		-0.002	-0.13 to 0.12		0.0003	-0.10 to 0.11	
Overall p value			0.17			0.0003			0.009
Linear term (overall effect)	0.01	-0.01 to 0.03	0.44	-0.01	-0.04 to 0.13	0.30	0.01	-0.007 to 0.04	0.19
Quadratic term	0.001	-0.002 to 0.004	0.51	-0.003	-0.01 to 0.002	0.23	0.002	-0.001 to 0.005	0.28

## Discussion

We studied working memory using the n-back, which is a widely used test of working memory, in a community based birth cohort. The n-back task mainly measures updating of working memory and consequently also involves executive function. This study is much larger than previous studies of working memory and *APOE* and thus has greater power to detect an effect of *APOE* genotype on working memory performance.

### Findings from our study

We found evidence to support reduced accuracy according to *APOE* genotype in the n-back at the 2-back level, but not the 3-back level though the direction of association was similar for 3-back. The adjusted multi-level model found evidence for an association with genotype, with worse performance in the ε22, ε 34 and ε44 groups. This supports our hypothesis that those with ε4 alleles would perform worse on the working memory task. We do not have any strong evidence to support an allele dose effect, but this may be due to the low numbers of ε44 homozygotes (n = 43). The ε22 group is also very small which may go some way towards explaining the worse performance in this group. The fact that the ε32 group did not perform worse strongly suggests that this is the likeliest explanation.

Previous studies have reported that ε2 possession is associated with better cognitive performance in old age, in contrast to our findings.[39, 40] Interestingly, neuropathological studies have suggested that this protection declines in old age, particularly after the age of 80.[41, 42] A recent fRMI study of middle aged adults found very similar activity patterns in the ε4+ and ε2+ groups during stroop and encoding tasks, with both groups showing increased activity in non-task related regions compared to the reference ε33 group. The authors commented that many previous fMRI studies had failed to adequately examine ε2 carriers and that the relationship between BOLD response and Alzheimer’s disease is probably not as simple as was first thought, with detriments possibly to be found in ε2 carriers as well as ε4 carriers. They also commented that the effects of APOE on functional connectivity needed further research.[43]

When reaction time was examined at each level separately there was evidence to support an effect of *APOE* genotype at both levels, although the evidence was slightly weaker for the 3-back. The adjusted multi-level model found evidence for an association with genotype, with faster reaction times in the ε32, ε34 and ε44 groups although only the ε34 group had a 95% confidence interval for the regression coefficient that did not cross zero. There were some conflicting results when reaction time was examined, however, with the ε44 group being slower than the reference group at the 3-back level, but faster at the 2-back level. It is notable that the ε44 group were less accurate at the 2-back level but not the 3-back. Reaction time is known to be a more variable measure than accuracy and it is because of this that accuracy is the usual primary outcome measure when assessing n-back performance. Because of the inconsistencies in this analysis we are therefore less confident that there is a genuine effect of genotype.

### Comparison with previous studies

In previous studies younger adults have been little studied, with most studies focusing on older adults. Several different working memory tasks, assessing different aspects of working memory have been used, making it difficult to compare study results. In one of two studies to include younger adults Alexander and colleagues studied 415 participants aged 6–65 and performed only the 1-back, finding no association of *APOE* genotype with working memory.[17] This may simply be a reflection of the low level of difficulty of the 1-back task and the fact that it is more of a measure of sustained attention. This study also had low numbers of ε44 (n = 13) although there were 91 ε4 carriers in total. They controlled only for age and education. Rusted and colleagues studied 41 young adults (aged 18–22 yrs) in a study which was primarily focused on fMRI. There was weak evidence of superior accuracy for ε4 carriers in the RVIP. Our study is therefore only one of two to study just young adults and is far larger than the study by Rusted et al.[13]

In the study with the next youngest cohort Reinvang and colleagues studied 186 40–80 year olds using the more difficult AX continuous performance task (participants had to detect a certain pair of letters). They found a deleterious effect of ε4 on working memory in male (but not female) ε44 homozygotes. [14] Given the low number of ε44 homozygotes and the subgroup analysis this may be a type 1 error. They did not control for co-variates. Greenwood and colleagues studied 177 healthy older individuals (41–85) from the BIOCARD study using a spatial working memory task. This study has robust methods but low numbers, did not control for covariates and no formal power calculation was given. They found a deleterious effect only in ε44 homozygotes (n = 12), particularly when the task required participants to remember 3 locations rather than 2.[15] Finally Deeny and colleagues studied 51 of older participants (50+) with a non-standard measure of working memory (cognometer battery). They found an adverse effect of ε4 on speed of processing in working memory tasks but not performance, after controlling for age, gender and education. [44, 45] Given the small size of these previous studies it seems entirely likely that low power and chance findings are a plausible explanation for the inconsistent findings. Our study used a well-recognised measure of working memory, was larger than the previous studies and did control for covariates

### Positive pleiotropy

Although the ε3 allele is now the most common it is thought that ε4 is the ancestral allele in humans. Interestingly other animals have only a single isoform of ApoE. It seems likely, given that the ε4 allele has persisted in humans, that there may be some positive effects in addition to its many deleterious effects. It may be, however, that because the deleterious effects mainly manifest after reproductive age that no such positive effects exist.

It was first suggested in 2001 by Hubacek that the ε4 allele may have positive pleiotropic effects. In a small study which randomly studied 1% of the population of a region in the Czech Republic they found that those with the ε4 allele were more likely to have attended higher education.[18] Later, larger studies have looked at IQ, educational attainment (e.g., SATS scores) and relation to *APOE* genotype and have not found any relationship prior to old age.[20, 46, 47] However, some authors have used evidence from functional imaging studies to supports the idea of positive pleiotropy in those with ε4 alleles.[23, 24] For example, Filippini et al found using BOLD fMRI that ε4 carriers had increased hippocampal activation during an encoding task but this could also be interpreted as a sign that more brain activation was required for a similar performance.[24] The exact mechanism of this positive pleiotropy, should it genuinely exist is unknown and no positive pleiotropic effects were seen in this study.

### Study Strengths and Limitations

Strengths of this study include the large sample size, adjustment for co-variates, the use of a more robust measure of working memory than some previous studies and the unselected nature of the sample. Despite this, the number of participants with ε44 was still quite low. Weaknesses include the lack of a 1-back as a measure of attention and the participants were slightly more intelligent than the general population. However we adjusted for a number of factors including IQ and still found differences according to genotype in working memory performance. This is a common problem in cohort studies where there is differential drop-out of those with lower socio-economic class and educational attainment.[48]

## Conclusions

In conclusion we have found evidence for a deleterious effect of ε44 *APOE* genotype on accuracy as a measure of working memory in a large birth cohort tested at age 18. This is consistent with the hypothesis that ApoE can affect neuronal repair early in life, well before the onset of the clinical signs and symptoms of dementia. It is possible that larger effects may be seen in older cohorts, perhaps as a result of the role of *APOE* in neuronal repair and the cumulative inefficiencies proposed in ε4 carriers. We found no evidence to support a positive pleiotropic effect of ε4.

## Supporting Information

S1 Fig The flow of participants through the study. (TIF)

S1 Table Accuracy and reaction time results for each genotype group adjusted for target. (DOCX)
