# Supplementary figures and images for: Possible Association of APOE Genotype with Working Memory in Young Adults
Source: PLoS One. 2015 Aug 19;10(8):e0135894. doi: 10.1371/journal.pone.0135894 (PMC4545585; doi:10.1371/journal.pone.0135894)

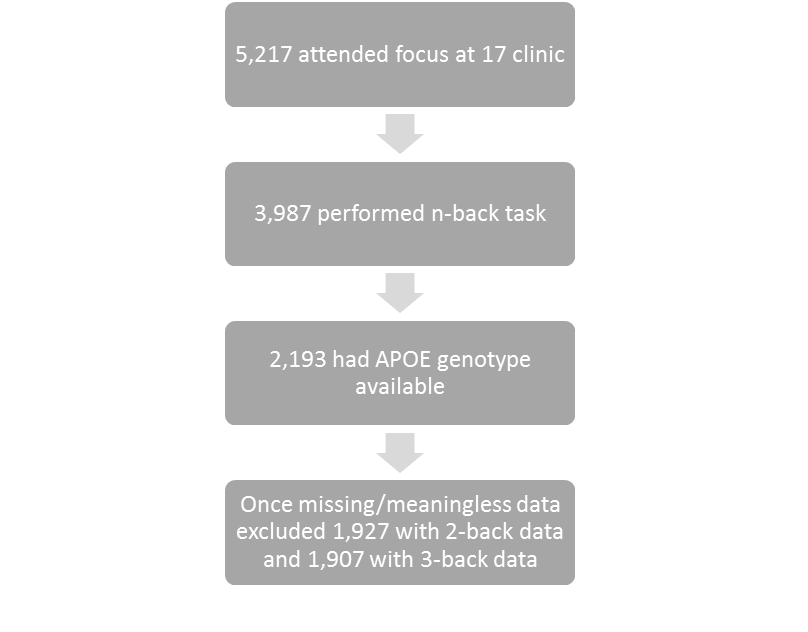

Supplement: S1 Fig — (TIF) [file pone.0135894.s001.tif]
