# Supplementary material for: Possible Association of APOE Genotype with Working Memory in Young Adults
Source: PLoS One. 2015 Aug 19;10(8):e0135894. doi: 10.1371/journal.pone.0135894 (PMC4545585; doi:10.1371/journal.pone.0135894)
Supplement: S1 Table — (DOCX) [file pone.0135894.s002.docx]

Supplementary table 1: Accuracy and reaction time results for each genotype group adjusted for target

|  | Mean Accuracy (SD)  Range 0-100% | | Median reaction time in milliseconds (SD) | |
| --- | --- | --- | --- | --- |
|  | 2 back | 3 back | 2 back | 3 back |
| ε22 | 74.97 (26.87) | 71.23 (21.55) | 700.57 (166.85) | 721.33 (194.34) |
| ε32 | 77.82 (21.99) | 67.22 (22.98) | 673.72 (222.70) | 705.57 (292.30) |
| ɛ33 | 76.92 (22.89) | 68.63 (22.78) | 693.96 (222.03) | 730.84 (291.05) |
| ε34 | 77.64 (21.85) | 68.66 (23.19) | 690.05 (220.31) | 725.47 (286.49) |
| ε44 | 70.66 (26.90) | 66.46 (25.18) | 725.02 (201.05) | 683.81 (207.22) |
